# Cholangiocarcinoma presenting as dysphagia and misdiagnosed as gastritis: a case report

**DOI:** 10.1186/s12876-022-02156-6

**Published:** 2022-02-23

**Authors:** Chao Wang, Baoyue Zhang, Runmei Peng, Zan Zuo, Hongzhong Cheng, Jun Zhu, Tianxing Chen, Zhengji Song

**Affiliations:** 1 Endoscopy Center, Panzhihua Central Hospital, Panzhihua, Sichuan China; 2 Medical School of Kunming University of Science and Technology, Kunming, Yunnan China; 3 Radiology Department, The First People’s Hospital of Yunnan Province, Kunming, Yunnan China; 4 Gastroenterology, The First People’s Hospital of Yunnan Province, Kunming, Yunnan China; 5 Cardio-Thoracic Surgery, The First People’s Hospital of Yunnan Province, Kunming, Yunnan China; 6 Department of General Surgery, The First People’s Hospital of Yunnan Province, Kunming, Yunnan China; 7 Pathology Department, The First People’s Hospital of Yunnan Province, Kunming, Yunnan China

**Keywords:** Cholangiocarcinoma, Gastric fundus, Cardia

## Abstract

**Background:**

Heterotopic tumor is a rare disease. Thus far, no cases of heterotopic cholangiocarcinoma have been reported in the world. Cholangiocarcinoma mainly metastasizes by direct invasion, and it can lead to liver metastasis in its advanced stage. There were few clinical cases of gastric metastasis in advanced tumors, mainly seen in breast cancer, lung cancer, liver cancer, malignant melanoma, choriocarcinoma, and hematological tumors. Metastases of cholangiocarcinoma to the stomach also are exceptionally rare.

**Case presentation:**

A 58-year-old man was admitted to the hospital because of difficulty swallowing for one year. Upon gastroscopy, we found the tumor at the region of the cardia and gastric fundus. Macroscopical appearance of the tumor suggested its malignant nature. Computed tomography (CT) findings showed that the wall of the cardia, fundus, and stomach body were thickened, suggesting a tumor. Because the patient had obvious difficulty swallowing, we invited cardiothoracic surgeons for consultation. They considered that the patient had definite mechanical obstruction in the lower esophagus; hence, they performed an operation. Immunohistochemical staining revealed low-to-medium differentiated adenocarcinoma (containing mucinous adenocarcinoma components) of biliary origin.

**Conclusions:**

We highlight the importance of the endoscopic biopsy of gastric tumor. However, when its results are inconsistent with the clinician’s judgment, further examination is required. Endoscopic ultrasonography and enhanced CT may be a good choice. If necessary, on the premise of patient acceptance, the diagnosis could be confirmed after surgical excision. Here we report a case of a patient with heterotopic cholangiocarcinoma in the gastric fundus. The most common tissue ectopias in the digestive tract include esophagogastric gastric mucosal ectopia, duodenal gastric mucosal ectopia, and gastric mucosal small intestinal ectopia. Thus far, there have been no reports of ectopic cholangiocarcinoma and associated cancer in the stomach. In addition, metastases of cholangiocarcinoma to the stomach are also exceptionally rare, and most of them are due to a direct invasion. The discovery of the primary lesion is an important clue for the reliable diagnosis in such cases.

## Background

Heterotopic tumor is a rare disease. Thus far, no cases of heterotopic cholangiocarcinoma have been reported. Patients with cholangiocarcinoma inside and outside the liver generally have abnormal liver function and symptoms of biliary obstruction, such as jaundice and itchy skin. Some patients may also have pain in the liver area [1]. The disease often has a high degree of malignancy [2, 3]. The main way of metastasis of cholangiocarcinoma is direct invasion, and liver metastasis can develop in the advanced stage. There have been few clinical cases of gastric metastasis in advanced tumors, mainly in breast cancer, lung cancer, liver cancer, malignant melanoma, choriocarcinoma, and hematological tumors. Metastases of cholangiocarcinoma to the stomach are exceptionally rare.

## Case presentation

A 58-year-old man was admitted to the hospital because of difficulty swallowing.

One year before the current admission, the patient noticed dysphagia when eating solid food, cough after drinking water, and belching. Since these symptoms were not getting worse and did not affect his daily life, the patient ignored them and did not ask for medical help.

Five months before the current admission, since the symptoms did not alleviate, the patient underwent gastroscopy at the outpatient clinic of another hospital. The gastroscopy report revealed a tumor at the region of the cardia and gastric fundus. The biopsy was obtained and showed signs of acute and chronic inflammation and active glandular hyperplasia at the gastric fundus. Since cancer was suspected, the physicians recommended obtaining another biopsy and transferring the patient to our hospital for further treatment, but the patient did not comply. He took omeprazole himself, but his symptoms never improved.

Eight weeks before the current admission, concerned with lack of significant improvement in his symptoms, the patient came to our hospital for the first time. The patient's weight dropped 3 kg compared with that in the time of his last visit of the clinic. On gastroscopy, we found a tumor at the region of the cardia and gastric fundus. Macroscopical appearance of the tumor suggested its malignant nature. Given that pathological results did not support cancer, the patient refused surgical exploration and requested to continue medical treatment and follow-up at the outpatient clinic. We instructed the patient to eat a liquid diet and take the following medications: esomeprazole enteric-coated tablets (20 mg *po bid*) to inhibit gastric acid secretion, almagate (1.5 g *po tid*) to protect gastric mucosa, and mosapride citrate (5 mg *po tid*) to promote gastrointestinal motility, and other methods for symptomatic treatment.

One week before this admission, the patient felt worsened dysphagia, with difficulty in eating a liquid diet, accompanied with nausea and vomiting. His weight was 5 kg lower than before [only 40.5 kg, Body mass index (BMI): 15.5 kg/m^2^]. Since the symptoms did not improve for a week, he revisited our hospital. We worried about the patient’s tumor progression. The patient was scheduled for gastroscopy and enhanced CT again, and a jejunal nutrition tube was placed using endoscopy. Because the patient had obvious difficulty swallowing, we invited cardiothoracic surgeons for consultation. They considered that the patient had definite mechanical obstruction in the lower esophagus; hence, they operated on the patient.

## Diagnostic Assessment

### Physical examination

Superficial lymph nodes throughout the body were not enlarged. Slight tenderness was present in the upper abdomen.

### Blood examinations

The patient underwent two successive blood examinations in our hospital (including blood cell test, liver enzymes, and tumor markers). Only one test result was obviously abnormal. Reference range of carbohydrate antigen 724 is 0.0–6.7 U/ml in our hospital. The patient's results were 17.9 U/ml (8 weeks before admission) and 67.77 U/ml (at the current admission). Reference range of alpha fetoprotein is 1.09–8.04 ng/ml in our hospital. The patient's results were 2.90 ng/ml (8 weeks before admission) and 3.13 U/ml (at the current admission). Reference range of carcinoembryonic antigen is 0.0–5.0 ng/ml in our hospital. The patient's results were 1.13 ng/ml (8 weeks before admission) and 1.58 U/ml (at the current admission). Reference range of carbohydrate antigen 199 is 0–37 U/ml in our hospital. The patient's results were 22.03 U/ml (8 weeks before admission) and 12.04 U/ml (at the current admission).

### Endoscopy

**Fig. 1:**
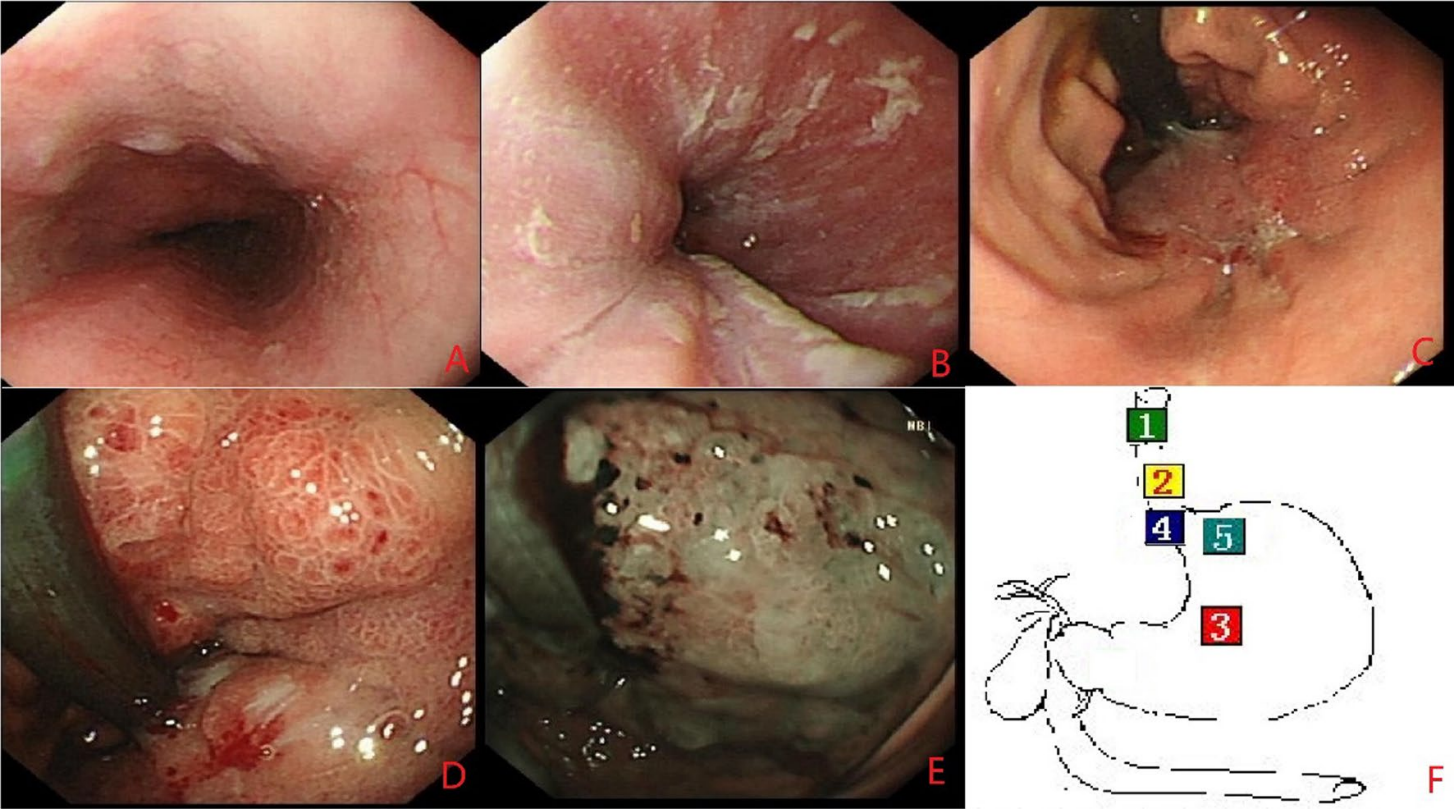
The first gastroscopy. **A** Middle section of the esophagus (approximately 30 cm from incisors). When entering the patient’s lower esophagus-cardia entrance (approximately 40 cm from incisors) (**B**), at this time, we had not found a tumor yet with sufficient steam injection. However, we failed to fully expose the squamous and columnar epithelial junction (SCJ). We turned the gastroscope over to look at the lesser curvature and found the lesion. First, gastric fundus dilation with sufficient gas injection was limited. Then, as shown in **C**, we found a bleeding spot in the cardia. Through closer observation, we found a lesion in form of a growing ring around the cardia. Because of its contraction, the stomach cavity was narrowed, similar to a gourd neck. The demarcation line (DL) of the lesion was clear, and as it was growing along the lesser curvature distally, the upper part of the stomach was also affected. Its mucosa was rough, protuberant, with spontaneous bleeding and prone to bleeding when touched by the endoscope. Surface structure was similar to papillary and granular. We did not see any obvious erosion, ulcer, or secretion. As shown in **D**, it seems to be a submucosal tumor with inflammatory changes in the surface mucosa. We switched to narrow band imaging (NBI) and could see that the lesion had DL. **E** We inserted biopsy forceps by gastroscopy to touch the lesion and verified that the texture of the lesion was stiff. **F** Position of the gastroscope lens in each panel of this figure

During the first gastroscopy, we found a lesion in form of a growing ring around the cardia. The boundary line of the lesion was clear, and it was growing along the lesser curvature distally. There were obvious mucosal swelling, spotty redness, and hyperemia on the surface of the lesion (Fig. 1). Our endoscopic diagnosis was the tumor in the gastric fundus and cardia requiring precise diagnosis (likely a nidus under the mucosa).

**Fig. 2:**
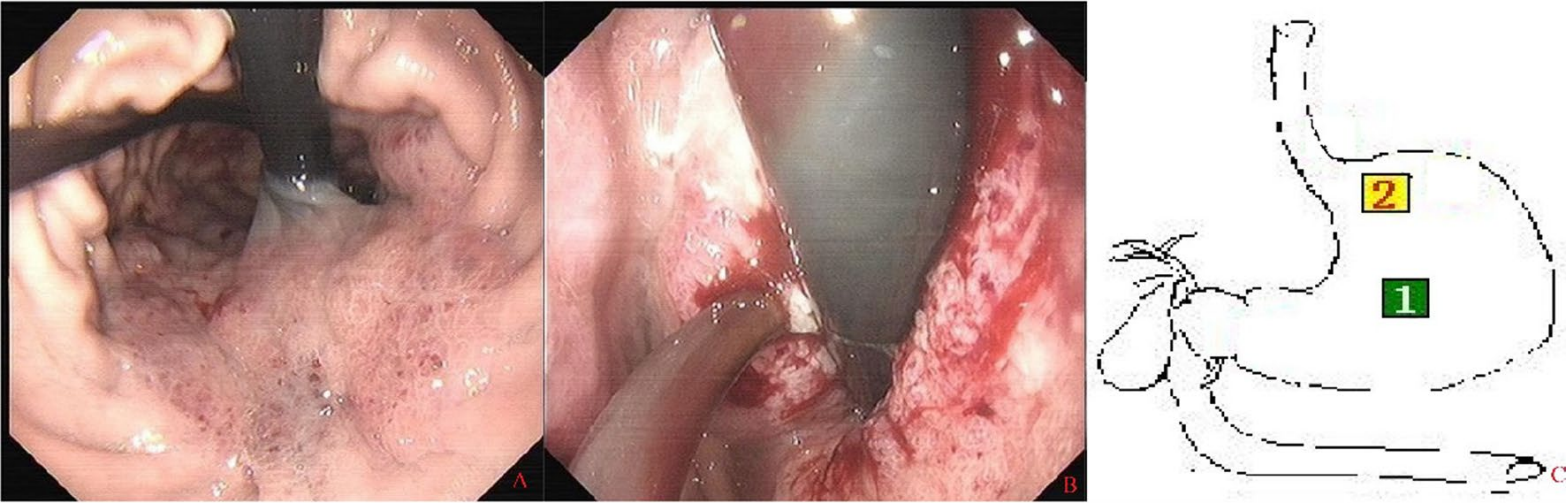
The second gastroscopy

During the second gastroscopy, we felt obvious resistance when passing the cardia, which may explain the reported worsening of dysphagia. When the gastroscope entered the stomach cavity, we found that the scope of the lesion had expanded since the last time; the most obvious was the spread to the far side, and rough mucosa of the lesion’s surface, erosion, and secretions could be seen (Fig. 2).

### Endoscopic ultrasonography

**Fig. 3:**
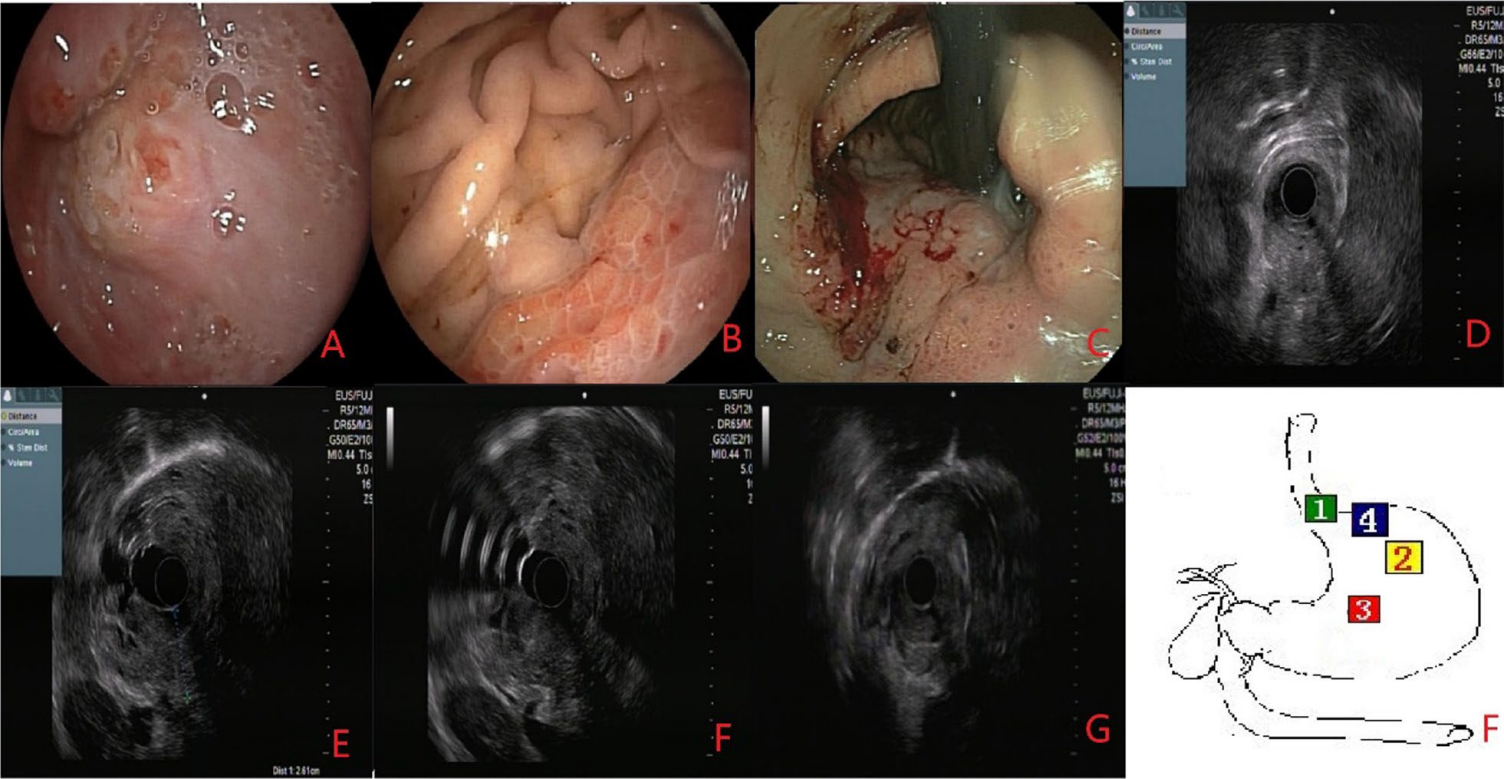
As shown in **A**, the mucosa at the SCJ was also partially congested and edematous. After the gastroscope passed through the cardia (**B**), it was evident that the lesion involved the fundus and greater curvature of the stomach. The diseased mucosa was congested, edematous, and bulged, resembling snakeskin. When we turned the gastroscope lens to observe the lesion closely (**C**), we observed the erosion and spontaneous bleeding. Endoscopic ultrasound showed thickened stomach wall, mucosal level disappearance, uneven echo of the lesion, manifested as low echo, and unclear boundary. The thickness of the thick part was about 2.6 cm, and the lesion invaded through the serosa, but we did not observe enlarged lymph nodes. The letters **A**–**C** in this figure correspond to the numbers 1–3 in **F**, and the letters **D**–**G** are all seen by ultrasound gastroscopy at the lesion, corresponding to the number 4 in **F**

Eight weeks before the current admission, we performed ultrasound gastroscopy to understand the source of the lesion and the depth of infiltration (Fig. 3). We used miniature probe endoscopic ultrasonography. Ultrasound gastroscopy offered the following diagnosis: the tumor of fundus-cardia (T3–T4 stage).

### Radiological examination

**Fig. 4:**
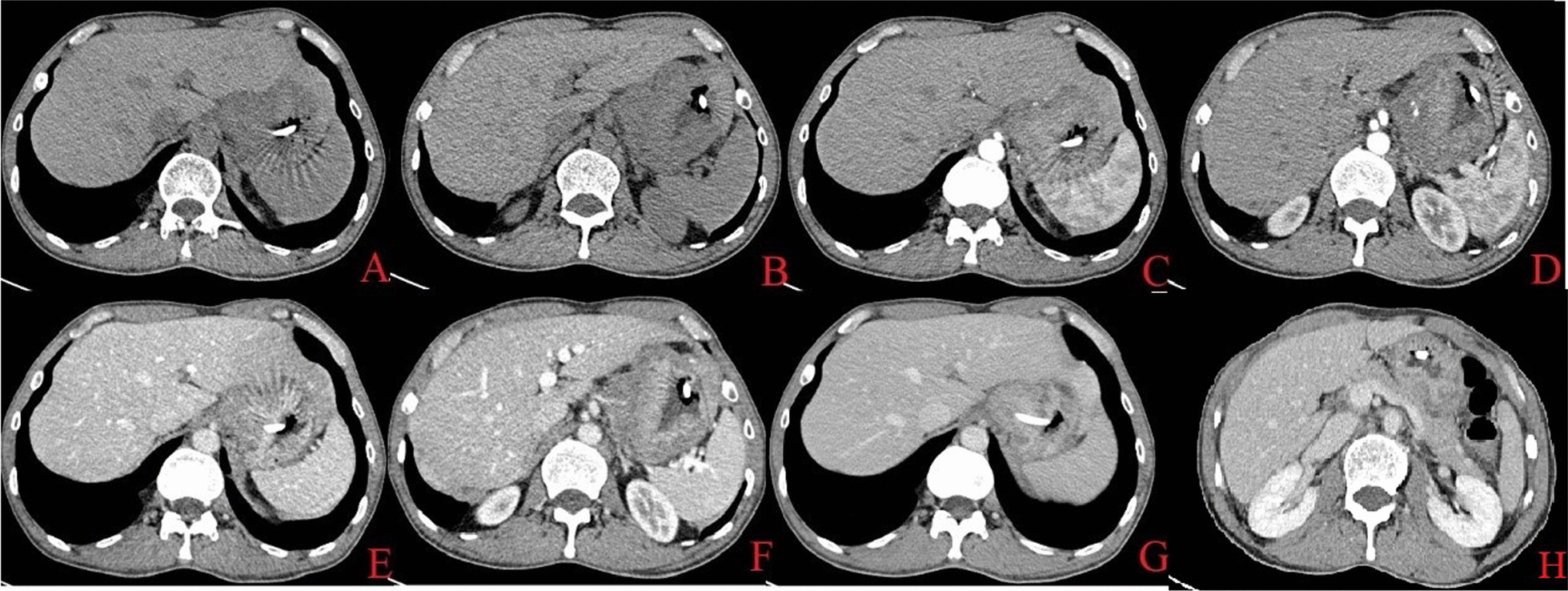
Enhanced CT images of the patient at the current admission (Siemens Force) CT plain and enhanced scan. Planes **A**, **B** plain scans, **C**, **D** arterial phase, **E**, **F** the portal vein phase, **G** the substantial phase, **H** image of extrahepatic bile duct. The indwelling nutritional tube shadow was seen in the stomach and esophagus. The thickening of the stomach wall was obvious, especially in the fundus and lesser curvature. On contrast-enhanced scan, the lesions showed heterogeneous enhancement. No obvious enlarged lymph nodes were found in the fat space around the lesion. There were no obvious dilatations of intra- and extrahepatic bile ducts. Imaging diagnosis showed that the gastric wall of the cardia, fundus, and stomach body were thickened, suggesting a tumor

Figure 4 shows the enhanced CT picture of the patient in this re-examination.

### Therapeutic intervention

#### Operation

**Fig. 5:**
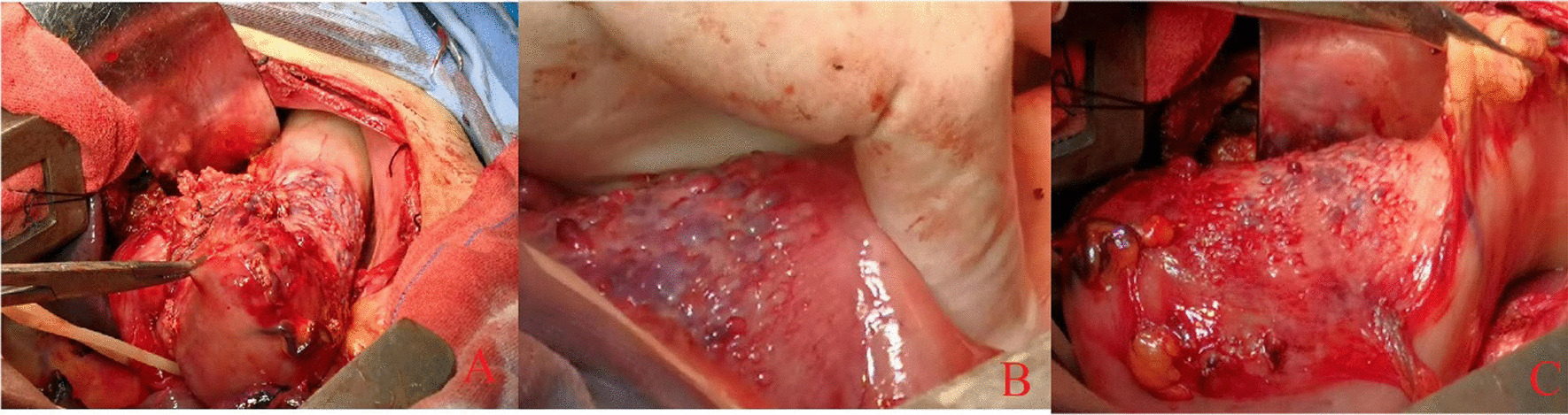
Intraoperative view

**Fig. 6:**
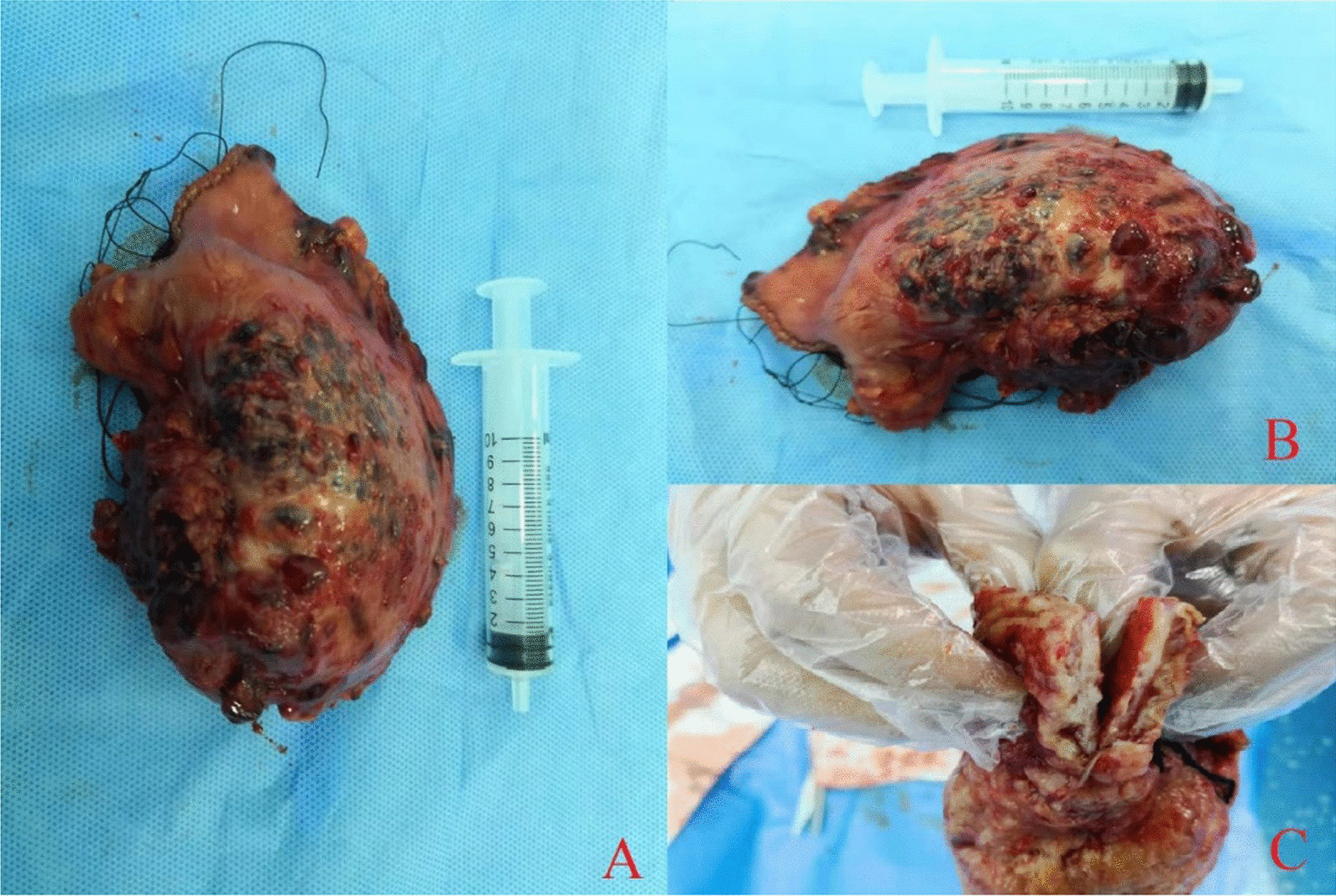
Gross surgically-excised specimens

Because the patient’s obstruction site was close to the lower esophagus, our surgical method was based on the transthoracic treatment method for the lower esophageal cancer. During exploration of the lungs and pleura of the left chest cavity, no obvious nodules were seen, and there were no enlarged lymph nodes near the esophagus. We continued to cut the diaphragm and esophageal hiatus about 8 cm, lifted the diaphragm, and observed swollen gastric lymph nodes near the cardia, as well as thickened, stiff and rough stomach wall at the fundus but without adhesions to the surrounding tissue. Figure 5 illustrates the view at surgical procedure. In Fig. 5A, we lifted the stomach wall at the lesion site with hemostatic forceps. The mass of the stomach wall could be moved. In Fig. 5B, the surface was uneven, and many blister-like protrusions of different sizes were visible on the surface. The local area was of a darker color and resembled the skin of a toad. The boundary between the lesion and the normal stomach wall was clear. In Fig. 5C, it shows the overall of the lesion. Considering that the patient had prominent gastric fundus lesions, for safety reasons, we invited a general surgeon (Dr Zhu Jun) to assist. Under his guidance, we removed the lesion with a disposable intraluminal cutting stapler and a disposable endoscopic cutting stapler, and anastomosed the esophagus with the distal stomach. The omentum and the pleura were suspended at the anastomosis to embed it. We sutured the stomach and diaphragm to fix the stomach. We placed a drainage tube between the sixth and seventh ribs. In Fig. 6A and B, the length of the lesion exceeds a 10ml syringe. In Fig. 6C, observation of the excised gastric tumor showed yellowish interstitial fluid exudation and many voids on the cut surface of the stomach wall, resembling a waffle. After the operation, the patient received intravenous drip of antibiotics for 3 days to prevent infection, the intravenous drip of omeprazole to inhibit the secretion of gastric acid, and an intramuscular drug to relieve pain. The patient was asked to fast and was given energy, water, and electrolytes by intravenous drip, water, and electrolytes.

Figure 6 shows the gross appearance of the excised specimen.

#### Pathohistology

**Fig. 7:**
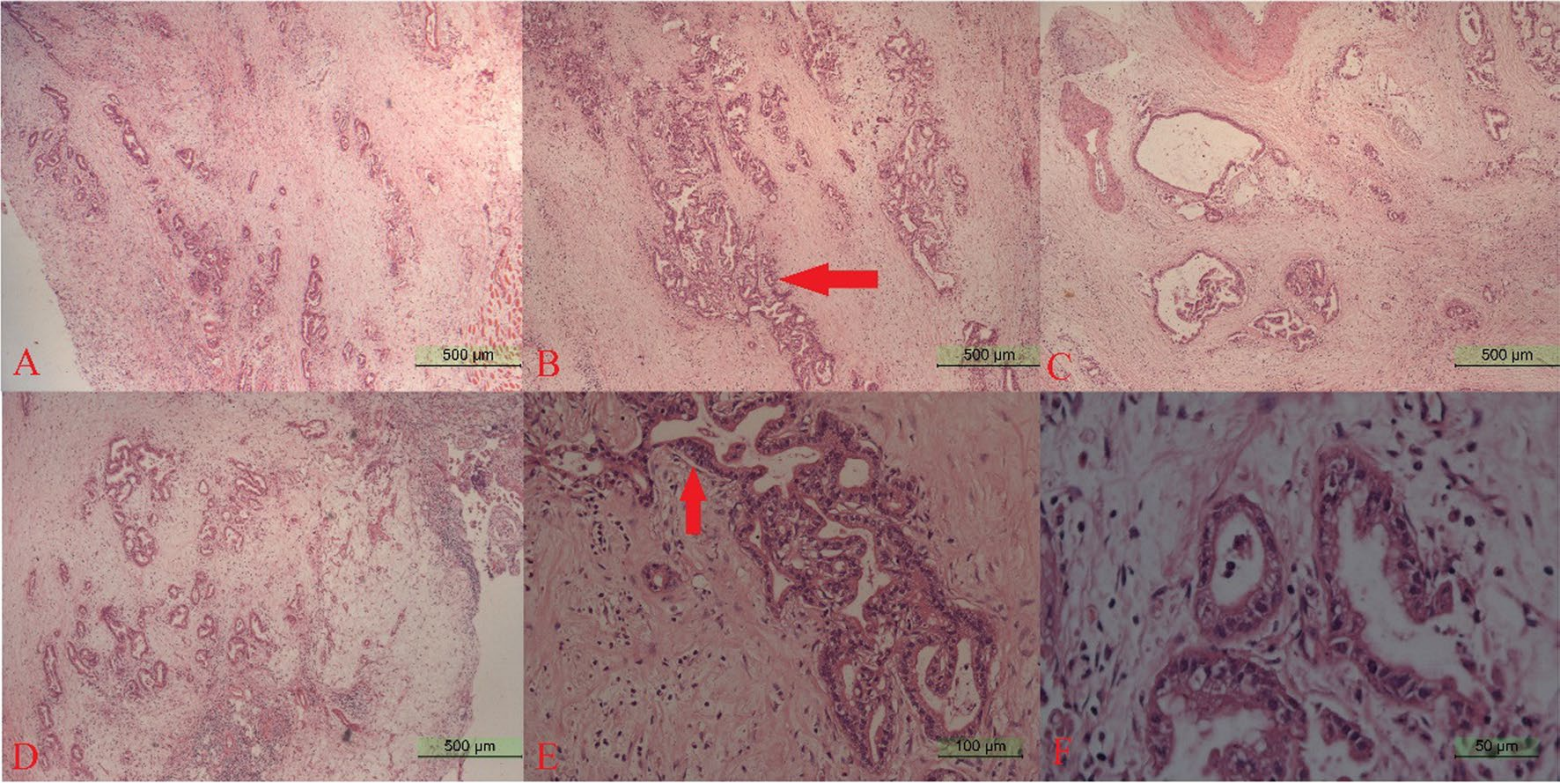
HE staining of the surgically excised lesions at this admission. The panels **A**–**D** show 5 × magnification, and the panels **E**, **F** show 20 × and 40 × magnifications, respectively. The entire sampling site is occupied by atypical bile duct epithelium. The tumor tissue is adenoid and the cells are atypical. Red arrows in the panels mark atypical bile ducts

Figure 7 shows the Hematoxylin–eosin (HE) staining of the surgically excised lesion. The pathohistological diagnosis was as follows: (most of the stomach) low-to-medium differentiated adenocarcinoma (including mucinous adenocarcinoma). Neural invasion was found, the serous layer was invaded, the two ends were negative, and the upper esophageal margin and the esophageal abdominal cavity were negative.

**Fig. 8:**
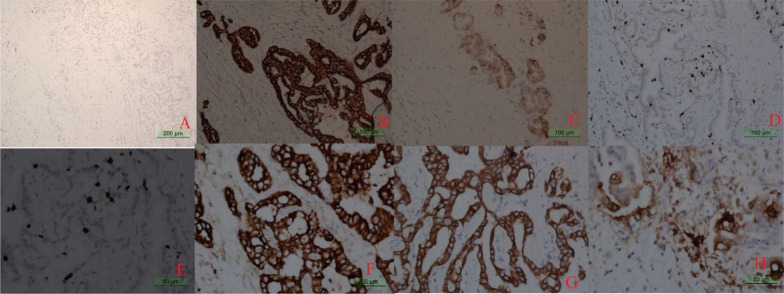
Immunohistochemical profile of the surgically excised lesions at this admission; the magnification is 10 × for CK20 in panel **A**, 20 × for LCK in panel **B**, 20 × for villin in panel **C**, 20 × for Ki-67 in panel **D**, 40 × for Ki-67 in panel **E**, 40 × for CK7 in panel **F**, 40 × for CK19 in panel **G**, and 40 × for CEA in panel **H**

The following immunohistochemical profile was obtained: (most of the stomach) Ki-67 (about 30%+), LCK (+), Vim (−), TTF-1 (−), HER-2 (weak positive), Hepa (local positive), PSAP (−), villin (+), CEA (local positive), CK19 (+), CK7 (+), CK20 (−), CDX-2 (focal positive). As shown in Fig. 8, immunohistochemical staining results support low-to-medium differentiated adenocarcinoma (containing mucinous adenocarcinoma components) of biliary origin.

#### Definite diagnosis

Heterotopic cholangiocarcinoma in the gastric fundus. The patient had no distant metastasis, and two lymph nodes metastasis in the postoperative specimens, So the stage of the tumor is the IIIA stage: T4aN1M0.

#### Follow-up and outcomes

Patients with cholangiocarcinoma inside and outside the liver generally have abnormal liver function and symptoms of biliary obstruction, such as jaundice and itchy skin. Some patients may also have pain in the liver area [1]. However, our patient’s onset site was in the stomach, and he only had difficulty swallowing. Postoperative pathohistological analysis confirmed cholangiocarcinoma, and although we did not find evidence of distant metastases, in view of high degree of malignancy of this disease [2, 3], we recommended continued chemotherapy to extend survival [4, 5]. However, the patient refused because of the cost of chemotherapy and died nearly five months after the operation.

## Discussion and conclusions

It has been reported that a constricting tumor at the gastroesophageal junction with probable invasion of the vagus nerves leads to features of achalasia and gastroparesis. The patient can also present with dysphagia [6, 7]. Such patients are without organic obstruction, but with signs of achalasia, such as beak sign on barium meal examination. Special manifestations may also appear during gastroscopy, including food residues in the esophageal cavity, dilatation of the esophageal cavity, rhythmic contraction rings in the esophagus, and resistance when endoscope passes through the cardia. However, the only manifestation in our patient on gastroscopy was that there was a sense of resistance when the gastroscope passed through the cardia. We considered it a sign of organic obstruction caused by the tumor compressing the cardia.

In fact, it is not uncommon to observe thickening of the stomach wall or gastric mucosa through endoscopy or CT. In such cases, we should consider most commonly *H. pylori* infection caused mucosal hypertrophy, followed by submucosal infiltration of poorly differentiated gastric cancer, and more rarely, gastric metastases, gastric lymphomas, Menetrier disease, Zollinger-Ellison syndrome, eosinophilic gastritis, gastric tuberculosis, and gastric sarcoidosis. Most of these diseases can be confirmed by endoscopic biopsy.

In the majority of the middle-aged and elderly patients, gastric cancer develops through atrophic gastritis-intestinal metaplasia-atypical hyperplasia-gastric cancer sequence, and *Helicobacter pylori* infection plays an important role in this process [8]. There have been reports of ectopic gastric mucosa in the bile duct [9], and even of the development of cholangiocarcinoma due to gastric mucosal ectopia [10]. We reviewed the relevant literature and did not find any reports of intragastric bile duct ectopia or intragastric cholangiocarcinoma. However, Japanese scholars have reported [11] a case of cholangiocarcinoma with intragastric metastasis after radical operation, indeed a very rare finding. By reviewing the two gastroscopic images of the patient, we can infer that the growth of this lesion at least started from the submucosa of the stomach and gradually infiltrated into the surface and serosa, which is similar to the growth process of gastric undifferentiated carcinoma. We have three assumptions about the etiology and pathogenesis of this tumor in our patient: The first possibility is based on ectopic intragastric bile duct cells. These abnormal cells turn into cancer cells after abnormal stimulation. The patient had a longer course of disease, combined with the normal bile duct appearance in CT and postoperative pathological findings of cholangiocarcinoma cells. So, we think that this assumption is most likely. The second possibility highlights that environmental factors may govern fully differentiated cells to undergo metaplasia and can cause such diseases, just like intestinal metaplasia. The third explanation may be that this was the metastasis of a cholangiocarcinoma to the stomach, although we think this option is less likely.

Finally, we highlight the importance of the endoscopic biopsy of the gastric tumor. However, when its results are inconsistent with the clinician’s judgment, further examination is required. Endoscopic ultrasonography and enhanced CT may be a good choice. If necessary, on the premise of patient acceptance, the diagnosis could be confirmed on surgically excised lesions. Here, we reported a case of a patient with heterotopic cholangiocarcinoma in the gastric fundus. The most common tissue ectopias in the digestive tract include esophagogastric gastric mucosal ectopia, duodenal gastric mucosal ectopia, and gastric mucosal small intestinal ectopia. So far, there have been no reports of ectopic cholangiocarcinoma and associated cancer in the stomach. In addition, metastases of cholangiocarcinoma to the stomach are exceptionally rare, and most of them are due to a direct invasion. The discovery of the primary lesion is an important clue for the reliable diagnosis in such cases.

## Data Availability

All available data are presented in the case.

## Abbreviations

CT: Computed tomography
BMI: Body Mass Index
SCJ: Squamous and columnar epithelial junction
DL: Demarcation line
NBI: Narrow band imaging
HE: Hematoxylin–eosin
